# Persistent thrombotic events in a seronegative patient with antiphospholipid syndrome

**DOI:** 10.1016/j.jdcr.2026.02.048

**Published:** 2026-03-10

**Authors:** Sabrina M. Saeed, Rebecca Fine, Hailey Baker, Fotios Koumpouras, Sarika Ramachandran

**Affiliations:** a Yale School of Medicine, New Haven, Connecticut; b Department of Dermatology, Yale School of Medicine, New Haven, Connecticut; c Section of Rheumatology, Allergy & Immunology, Department of Internal Medicine, Yale School of Medicine, New Haven, Connecticut

**Keywords:** anticoagulation, antiphospholipid syndrome, seronegativization

## Background

Antiphospholipid syndrome (APS) is a systemic autoimmune disorder characterized by arterial, venous, or small vessel thromboses in the presence of antiphospholipid antibodies detected by assays for lupus anticoagulant activity (LA) and antibodies to cardiolipin, and beta-2 glycoprotein I.

The standard of care for APS involves anticoagulation with warfarin or other vitamin K antagonists, while use of direct oral anticoagulants (DOACs) in this population remains controversial.

Seronegativization, where previously detected antiphospholipid antibodies become undetectable, has been reported in patients with an incidence of 9% to 59%.^1^, ^2^, ^3^, ^4^ This phenomenon may happen spontaneously or as a result of therapy and is more common in patients positive for only 1 antibody.^1^^,^^2^ The clinical implication of an unstable profile remains unclear, including whether anticoagulation can be safely discontinued after achieving seronegativity.

Here, we present the case of a patient with APS who experienced persistent thrombotic events despite continuous anticoagulation and seronegative status.

## Case

A 66-year-old male with a history of coronary artery disease, atrial fibrillation, peripheral vascular disease, type 2 diabetes mellitus, and stage 4 chronic kidney disease was transferred from a referring institution with a necrotic skin lesion on the right lateral chest wall. Seven years prior to presentation, the patient experienced an unprovoked pulmonary embolism and was found to have elevated anti-beta-2 glycoprotein I IgG (53.7, normal <9) and IgA (37.3, normal <9) which remained elevated after 12 weeks, thus meeting the revised Sapporo criteria for APS.^5^ Anticardiolipin antibodies and lupus anticoagulant were not elevated on multiple occasions. The patient was initially anticoagulated with warfarin but transitioned to apixaban several years before the current admission. During this period, APS-related laboratory markers underwent seronegative conversion, remaining persistently negative at 15 months and 6 years after APS diagnosis.

**Fig 1:**
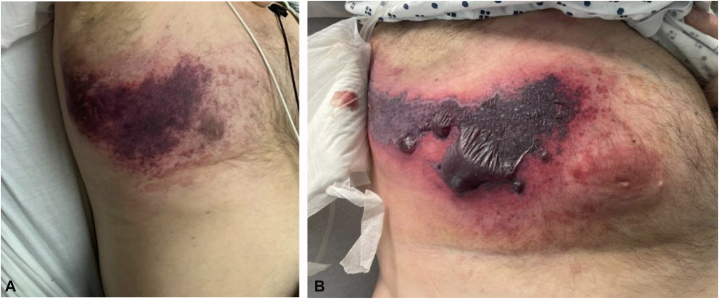
**A,** Initial presentation with purpuric patch over the right lateral chest wall. **B,** Retiform purpuric patch on right chest wall 4 days later, upon transfer to the tertiary care center.

**Fig 2:**
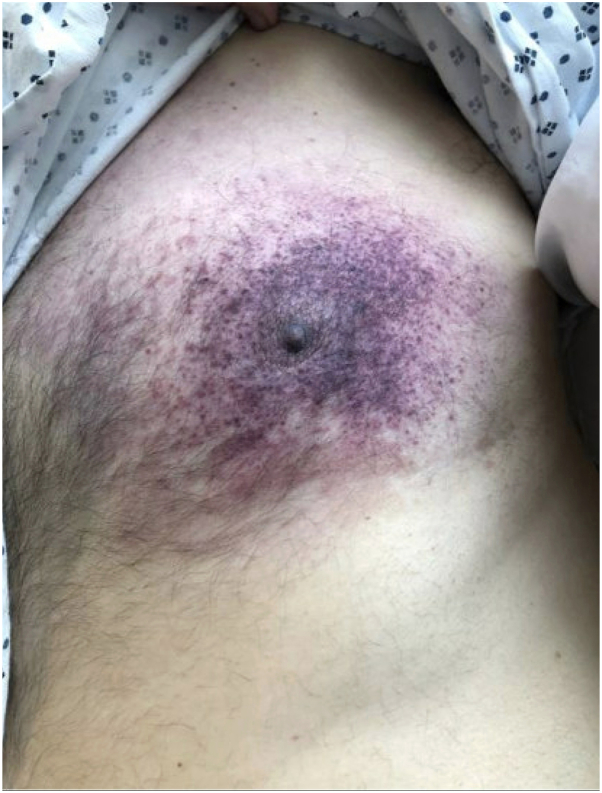
Purpura over the left chest.

**Fig 3:**
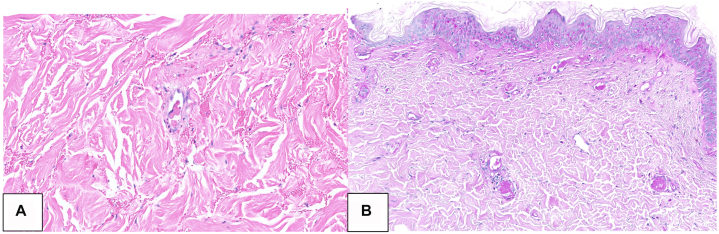
Pathology of right chest lesion demonstrating superficial to deep dermal small vessels with congestion and eosinophilic deposits within them (hematoxylin-eosin stain, ×400) (**A**). PAS stain highlights the intravascular deposits (PAS stain, ×100) (**B**).

One week before transfer to our institution, he was hospitalized with a purpuric patch on the right lateral chest which developed acutely after weightlifting (Fig 1, *A*). Out of concern for a hematoma, his apixaban was held. Laboratory evaluation revealed elevations in erythrocyte sedimentation rate (54 mm/h), creatinine (3.22 mg/dL), calcium (9.7 mg/dL), and D-dimer (19, normal <0.5 mg/mL), with normal hemoglobin (12.1 g/dL), platelets (177 K/uL), PT (11.1 seconds), and PTT (26.3 seconds). Protein C, Protein S, antithrombin III, and cryoglobulin levels were within normal limits. A CT angiogram of the upper extremity revealed atherosclerotic vascular disease with mild aneurysmal dilatation of the proximal right subclavian artery measuring 1.4 cm maximally without evidence of dissection or hematoma. Due to worsening skin findings, he was transferred to our tertiary care center. Physical examination demonstrated a retiform purpuric plaque on the right lateral chest with angulated borders, a hemorrhagic bullae, and surrounding erythema (Fig 1, *B*) and a purple macule on the dorsal left second toe. Urinalysis was positive for 2+ protein, 4+ glucose, and trace blood. While anticoagulation was held, he developed new purpura over the previously unaffected left breast (Fig 2). Punch biopsy of the right lateral chest revealed micro-occlusive vasculopathy with intravascular PAS-positive material and epidermal and dermal necrosis without significant inflammatory infiltrate (Fig 3). Tissue and blood cultures were negative.

**Fig 4:**
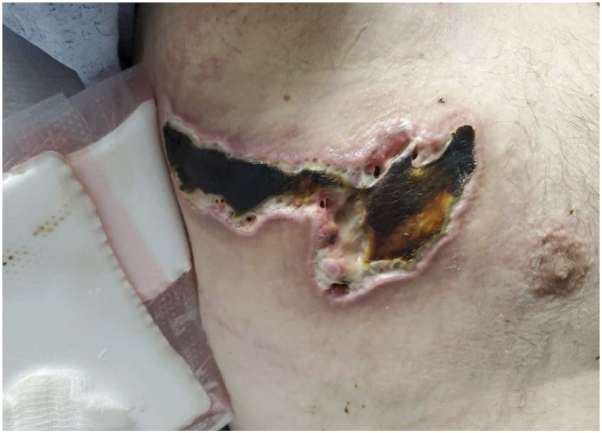
Necrotic lesion 6 weeks after initial presentation.

Due to the patient’s history of APS, he was anticoagulated with heparin. Laboratory tests for ANCA antibodies, anti-beta-2 glycoprotein I, anticardiolipin and lupus anticoagulant (tested while off anticoagulation) were found to be negative. Despite seronegativity, his presentation was attributed to APS based on biopsy findings and clinical history. The patient was transitioned from heparin drip back to warfarin. Due to the rapid progression of the initial lesion, new involvement of the left chest, and identification of a nonocclusive thrombus in the left popliteal, he was treated with prednisone and IVIG, though ultimately felt not to meet criteria for catastrophic antiphospholipid syndrome. He underwent wound debridement (Fig 4) and vacuum-assisted closure with full healing 5 months after initial presentation.

## Discussion

This case underscores the challenges of diagnosing and managing APS in patients with complex comorbidities and highlights the unclear risk of recurrent thrombosis in patients with APS who exhibit an unstable aPL antibody profile.

Preventing recurrent thrombotic events is the main goal of therapy in APS. Although DOACs offer predictable pharmacokinetics, no requirement for routine monitoring, and fewer drug interactions, several trials have demonstrated elevated clotting risk compared to warfarin.^2^, ^3^, ^4^, ^5^ DOACs are therefore often reserved for uncomplicated cases of APS or for patients who cannot tolerate warfarin or LMWH. Current EULAR guidelines recommend against the use of DOACs in high-risk APS patients with arterial thrombosis and triple positivity for APL antibodies.^6^ Our case further confirms that DOACs are insufficient for patients with multiple thrombotic risk factors such as severe atherosclerotic disease, underscoring the necessity for more robust anticoagulation in high-risk patients. Whether anticoagulation can be safely discontinued in APS patients after antibody negativization remains unclear, with varying evidence on the occurrence of thrombotic events in seronegative patients. One study reported pulmonary embolism in 1 out of 10 seronegative patients within 19-months off of anticoagulation,^7^ while another study reported no thrombotic events in 13 of 55 individuals with SLE and APS who discontinued anticoagulation.^8^ Other studies suggest higher thrombosis rates. Continued thrombotic risk may be multifactorial. While LAC, anti-aCL, and anti-B2GP1 are the main antibodies implicated in APS, additional procoagulant “noncriteria” autoantibodies exist.^6^ Additionally, aPL contributes to oxidative stress, inflammation, and genomic and epigenetic changes which persist beyond seronegativization. Lastly, comorbid metabolic and cardiovascular risk factors may increase risk of recurrence. In 1 study, 46% of primary APS patients with subsequent negative aPL developed thrombosis despite being on warfarin, with most of these patients also having concomitant cardiovascular risk factors.^9^ In 2022, a DELPHI consensus statement formally defined seroconversion as 2 consecutive negative aPL values 1-year apart^10^ and recommended continued anticoagulation for patients with a history of unprovoked thrombosis and ongoing risk factors. For patients with provoked DVT and resolved risk factors, VKA suspension could be considered, especially in single aPL-positive individuals without underlying connective tissue diseases and with a plan for prophylaxis in high-risk situations and regular follow-up visits. Ultimately, while seronegativization may suggest a reduced risk of thrombosis, anticoagulation in patients with multiple cardiovascular risk factors should be carefully individualized, accounting for the overall risk-benefit profile.

## Conflicts of interest

None disclosed.
